# Myocardial involvement and deformation abnormalities in idiopathic inflammatory myopathy assessed by CMR feature tracking

**DOI:** 10.1007/s10554-020-02020-2

**Published:** 2020-09-17

**Authors:** Johannes Kersten, Ahmet Muhammed Güleroglu, Angela Rosenbohm, Dominik Buckert, Albert Christian Ludolph, Carsten Hackenbroch, Meinrad Beer, Peter Bernhardt

**Affiliations:** 1 Department of Internal Medicine II, University of Ulm, Albert-Einstein-Allee 23, 89081 Ulm, Germany; 2 Heart Clinic Ulm, Magirusstr. 49, 89077 Ulm, Germany; 3 Department of Neurology, University of Ulm, Oberer Eselsberg 45, 89081 Ulm, Germany; 4 Department of Radiology, Armed Forces Military Hospital Ulm, Oberer Eselsberg 40, 89081 Ulm, Germany; 5 Department of Diagnostic and Interventional Radiology, University of Ulm, Albert-Einstein-Allee 23, 89081 Ulm, Germany; 6 University Hospital of Ulm, Albert-Einstein-Allee 23, Ulm, Germany

**Keywords:** Idiopathic inflammatory myopathies, Cardiac magnetic resonance, Late gadolinium enhancement, Strain imaging

## Abstract

**Background:**

Cardiac involvement has been described in idiopathic inflammatory myopathies (IIM), including non-specific ECG and echocardiographic findings. Aim of our study was to evaluate myocardial deformation parameters in IIM and to correlate them with late gadolinium enhancement (LGE) findings using cardiac magnetic resonance imaging (CMR).

**Methods:**

Forty-seven consecutive patients with histologically proven IIM were included into our study. Twenty-five healthy volunteers were used as a control group. All patients and controls underwent CMR examination using a 1.5 T scanner including functional cine and LGE imaging. After a mean follow-up of 234.7 ± 79.5 days a second CMR examination was performed in IIM patients.

**Results:**

In comparison to healthy volunteers, IIM patients had lower left ventricular mass and left ventricular global radial, circumferential and longitudinal strain. There was no significant difference in left ventricular ejection fraction.

Patients with LGE (N = 28) had lower left ventricular ejection fraction (p = 0.016), global right and left ventricular longitudinal strain (p = 0.014 and p = 0.005) and global left ventricular diastolic longitudinal strain rate (p = 0.001) compared to patients without LGE (N = 19).

In IIM patients, a significant decrease of left ventricular ejection fraction, left ventricular mass and all measured deformation parameters was observed between baseline and follow-up CMR.

**Conclusion:**

Cardiac involvement in IIM is frequent. Impairment of systolic and diastolic deformation parameters and a worsening over time can be observed. CMR is a useful tool for cardiac diagnostic work-up of these patients.

## Introduction

Idiopathic inflammatory myopathies (IIM) are potentially treatable chronic autoimmune disorders with muscle weakness, muscle enzyme elevation, and extra-muscular manifestations [1, 2]. They include different well-characterized disorders including dermatomyositis, polymyositis, necrotizing autoimmune myositis, inclusion-body myositis and overlap syndrome [2]. Cardiac involvement is a common finding in these patients and is reported in about 32 to 77% [3]. In comparison to the general population, patients with IIM have an increased risk for cardiac death [4]. Thereby, an early detected cardiac involvement of IIM could have influence on therapeutic regime leading to improvement of survival [5]. Accordingly, the Working Group on Myocardial and Pericardial Diseases of the European Society of Cardiology detected a lack in diagnostic work-up for patients with inflammatory diseases [6]. Classical physical stress tests and laboratory tests are prone to error in IIM patients because of muscular fatigue and muscular isoenzyme interactions [7].

Cardiac magnetic resonance (CMR) imaging has become the standard non-invasive imaging modality in inflammatory myocarditis [8] and for detection of cardiac involvement in systemic muscle diseases [9–11]. It has also been shown to be a valuable tool for detection of cardiac involvement in patients with IIM [10, 11]. With modern methods of feature tracking, measurement of myocardial deformation has shown its prognostic value in different cardiac entities [12, 13]. Aim of our study was to evaluate myocardial deformation parameters in IIM and their development over time. Deformation parameters were correlated with Late gadolinium enhancement (LGE) findings and compared with an age and gender matched control group using cardiac magnetic resonance imaging.

## Materials and methods

### Study population

Patients with known and treated IIM and patients presenting with suspected inflammatory myopathy were screened for inclusion into the study. Before enrollment the diagnosis had to be histopathologically proven by skeletal muscle biopsy. CMR-Scans were performed at baseline and after a follow-up of about 6 to 9 months. A control group of healthy volunteers were screened for age and gender match.

Exclusion criteria were contraindication for CMR, gadolinium-based contrast agent, pregnancy, unknown contraception or a severely reduced condition due to immobility because of severe paresis or the diagnose of inclusion body myositis. All included patients gave informed written consent. The study was approved by the local ethics committee (approval no. 13/10).

### Cardiac magnetic resonance

#### Image acquisition

CMR was performed in all patients using a 1.5T scanner (Intera, Philips Medical Systems, Best, Netherlands) with a 32-channel phased-array cardiac surface coil. For volumetric, functional, and feature tracking analysis a steady-state free precession cine sequence (repetition time 3.4 ms, echo time 1.7 ms, slice thickness 8 mm, no interslice gap, acquisition in end-expirational breath-hold) in contiguous short axis orientation covering the entire left and right ventricle, and three long axis views (2-, 3- and 4-chamber view orientation) were obtained as previously reported [11, 14].

For LGE imaging, gadoterate meglumine (Dotarem ®, Guerbet, Villepinte, France) was administered intravenously at the dose of 0.2 mmol/kg body-weight. After 10 min, a look-locker sequence for individual adjustment of the inversion time was performed in short axis orientation. Thereafter, an inversion-recovery gradient-echo sequence (repetition time 7.1 ms, echo time 3.2 ms, slice thickness 8 mm, respiratory navigator) for evaluation of LGE was acquired in the same orientations as the cine images [11, 14].

#### Image analysis

All CMR images were anonymized before DICOM transfer to a dedicated workstation. Two experienced readers, blinded to the clinical data, evaluated all images in consensus using a dedicated software (cmr42, Circle, Calgary Canada). Cine images were analyzed for right and left ventricular end-diastolic, end-systolic volumes, and for left ventricular myocardial mass. Ejection fractions were calculated, correspondingly. Deformation parameters were evaluated using feature tracking.

**Fig. 1:**
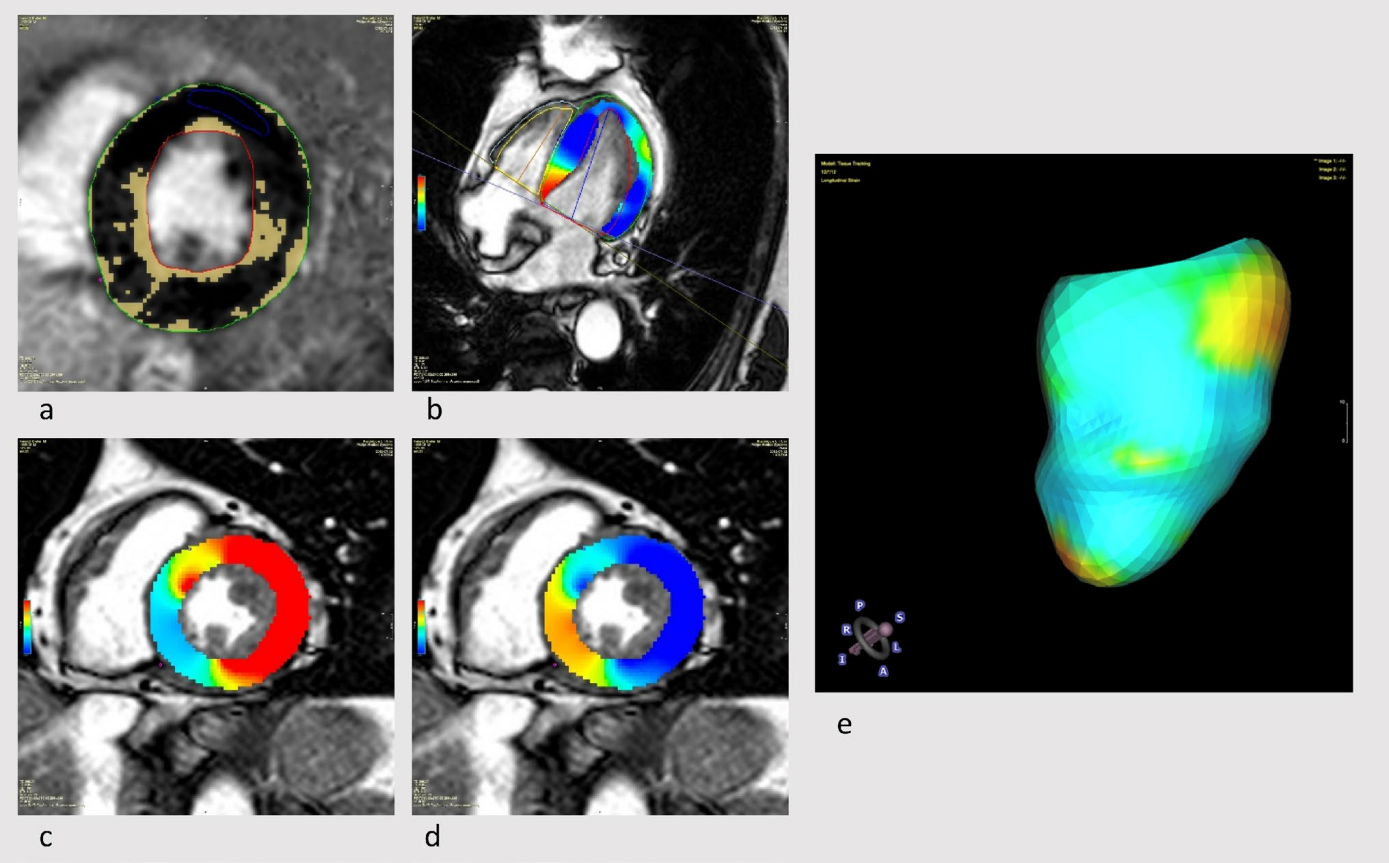
Patient with idiopathic inflammatory myopathy. **a** Short axis view of a late gadolinium enhancement sequence (yellow colored parts with pathological enhancement), **b** long axis four-chamber view in end-systole with illustration of the longitudinal strain, **c** and **d** short axis view in end-systole with illustration of radial (**c**) and circumferential strain **d**, **e** 3D-reconstruction of the left ventricle colored according to the longitudinal strain

The inversion-recovery gradient-echo sequence was evaluated for presence of LGE by both readers in consensus. Subepicardial and/or intramural LGE was defined as non-ischemic, whereas subendocardial and/or transmural LGE as ischemic. An example for left ventricular LGE-imaging and of the visualization of myocardial deformation of a patient with IIM is seen in Fig. 1.

### Statistical analysis

All data are reported as mean value and standard deviation. In case of normally distribution in the Shapiro-Wilk test, the Student *t* test for normally distributed variables was used. Otherwise, Mann-Whitney-U test was performed. A p-value < 0.05 was regarded to be statistically significant.

## Results

### Study population

The study group consisted of 53 patients with histologically proven idiopathic inflammatory myopathy. In all these patients basline CMR After a mean follow-up of 234.7 ± 79.5 days in 47 patients, the second CMR scan could be performed. There was a loss of follow-up in 6 patients. In the study group, 10 patients had dermatomyositis, 31 had polymyositis and 6 had other subforms of IIM. Twenty-five healthy, age and gender matched volunteers underwent CMR examinations as control group. Baseline characteristics and general findings of the study and control groups are illustrated in Table 1. In the study cohort, 19 patients were hypertensive (none in control group), 8 had dyslipidemia (2 in control group), 2 had diabetes mellitus and 8 were smokers (both none in control group). Neither patients, nor controls had a known history of myocardial disease at baseline.

**Table 1:** Baseline characteristics and general results of patients with Idiopathic inflammatory myopathy (IIM) and Controls

	Patients with IIM (N = 47)	Control group (N = 25)	p-value
Age (years)	54.3 ± 15.4	53.4 ± 10.75	0.771
Males (n)	22 (46.8%)	12 (48.0%)	0.924*
BMI (kg/m^2^)	26.5	25.1	0.861
Hypertension (n)	19 (40.4%)	0 (0%)	< 0.001*
Type 2 diabetes (n)	8 (17.0%)	2 (8%)	0.512*
Dyslipidemia (n)	2 (4.3%)	0 (0%)	0.030*
Smoker (n)	8 (17.0%)	0 (0%)	0.030*
LVEF (%)	64.4 ± 5.8	62.0 ± 5.6	0.102
LVEDVi (ml/m^2^)	76.5 ± 16.9	70.8 ± 11.1	0.141
LV mass (g)	90.2 ± 28.5	102.0 ± 28.5	0.102
LV mass/LVEDVi (g/ml/m^2^)	1.195 ± 0.31	1.448 ± 0.35	0.003
RVEF (%)	63.5 ± 5.9	58.8 ± 6.9	0.003
RVEDVi (ml/m^2^)	66.3 ± 15.4	66.7 ± 14.0	0.923
LVGRS (%)	36.8 ± 7.0	42.4 ± 7.6	0.003
LVGCS (%)	− 18.8 ± 2.4	− 20.9 ± 2.4	< 0.001
LVGLS (%)	− 17.4 ± 2.4	− 20.1 ± 2.2	< 0.001

*Mann-Whitney-U test used

### CMR results

All CMR scans were performed without complications. Left ventricular function and volumes were normal in all patients (with LVEF > 55% considered as normal). In comparison to our healthy control group, IIM patients had similar LVEF (64.4 ± 5.8%vs. 62.0 ± 5.6%, p = 0.102) and left and right ventricular volumes. In difference, they had a statistically reduced strain parameters, including left ventricular global radial strain (LVGRS) (36.8 ± 7.0% vs.42.4 ± 7.6%, p = 0.003), left ventricular global circumferential strain (LVGCS) (− 18.8 ± 2.4 vs. − 20.9 ± 2.4, p < 0.001) and left ventricular global longitudinal strain (LVGLS) (− 17.4 ± 2.4% vs. − 20.1 ± 2.2%, p < 0.001). There was also a non-significant reduction in left ventricular mass (90.2 ± 28.5 g vs. 102.0 ± 28.5 g, p = 0.102), which reaches significance when reported to the LVEDVi (left ventricular mass/LVEDVi) (1.448 ± 0.35 g/ml/m^2^ vs. 1.195 ± 0.31 g/ml/m^2^, p = 0.003). There were significant higher scores for right ventricular ejection fraction (RVEF) in the study-group than in controls (63.5 ± 5.9 vs. 58.8 ± 6.9, p = 0.003).

A pathological LGE was found in 28 patients, while the LGE pattern was always non-ischemic. In comparison, patients without LGE had higher values for LVEF, LVGLS, right ventricular global longitudinal strain (RVGLS) and left ventricular longitudinal diastolic strain rate. There were no significant differences in other volumetric or deformation parameters (see Table 2). In one case, a new pathological LGE was found at follow up.

**Table 2:** Volumetric and strain parameters of patients with idiopathic inflammatory myopathies (IIM), comparison between patients with and without late gadolinium enhancement (LGE)

	IIM without LGE (n = 19)	IIM with LGE (n = 28)	p-value
LVEF (%)	69.4 ± 4.9	64.8 ± 6.4	0.016
LVEDVi (ml/m^2^)	77.5 ± 15.9	75.0 ± 16.2	0.612
LV mass (g)	106.3 ± 36.1	89.9 ± 27.9	0.095
RVEF (%)	65.2 ± 6.3	62.5 ± 5.5	0.134
RVEDVi (ml/m^2^)	70.1 ± 14.2	66.5 ± 13.2	0.397
LVGRS (%)	41.3 ± 7.1	38.9 ± 7.1	0.254
LVGCS (%)	− 20.0 ± 2.0	− 19.7 ± 2.5	0.662
LVGLS (%)	− 19.7 ± 2.2	− 17.8 ± 2.2	0.005
RVGLS (%)	− 31.0 ± 2.8	− 28.3 ± 4.0	0.014
Left ventricular systolic longitudinal strain rate (/sec)	− 1.161 ± 0.24	− 1.093 ± 0.26	0.365
Left ventricular diastolic longitudinal strain rate (/sec)	1.266 ± 0.25	1.063 ± 0.24	0.008

**Fig. 2:**
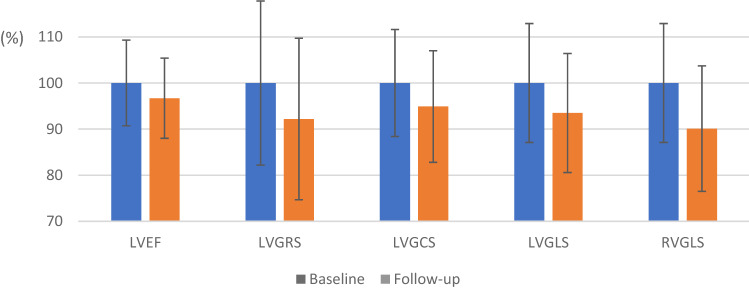
Relative reduction of left ventricular ejection fraction (LVEF), left ventricular global radial strain (LVGRS), left ventricular global circumferential strain (LVGCS), left ventricular global longitudinal strain (LVGLS) and right ventricular global longitudinal strain (RVGLS) from baseline to follow-up

At follow-up, every systolic functional parameter was reduced. Whereas the relative reduction of LVEF was modest but significant (66.6% ± 6.2 to 64.4% ± 5.8, p = 0.006), the decrease in deformation parameters including LVGRS, LVGCS, LVGLS and RVGLS was more substantial (see Fig. 2). There was also a reduction in left ventricular systolic longitudinal strain rate (− 1.12 /s ± 0.25 to − 1.03 /s ± 0.25, p = 0.011) and left ventricular diastolic longitudinal strain rate (1.15 /s ± 0.26 to 0.95 /s ± 0.24, p < 0.001). Left ventricular mass was reduced at time of follow-up (96.1 g ± 31.8 to 90.2 g ± 28.5, p = 0.004). There was no significant difference in left or right ventricular volumes or RVEF (see Table 3).

**Table 3:** Volumetric and strain parameters in patients with idiopathic inflammatory myopathies (IIM), comparison between baseline and follow-up measurements

	IIM at baseline (N = 47)	IIM at follow-up (N = 47)	p-value
LVEF (%)	66.6 ± 6.2	64.4 ± 5.8	0.006
LVEDVi in (ml/m^2^)	76.0 ± 15.9	76.5 ± 16.9	0.751
LV mass (g)	96.1 ± 31.8	90.2 ± 28.5	0.004
RVEF (%)	63.5 ± 5.9	62.4 ± 5.8	0.136
RVEDVi (ml/m^2^)	67.8 ± 13.5	66.3 ± 15.4	0.184
LVGRS (%)	39.9 ± 7.1	36.8 ± 7.0	< 0.001
LVGCS (%)	− 19.8 ± 2.3	− 18.8 ± 2.4	< 0.001
LVGLS (%)	− 18.6 ± 2.4	− 17.4 ± 2.4	< 0.001
RVGLS (%)	− 29.4 ± 3.8	− 26.5 ± 4.0	< 0.001
Left ventricular systolic longitudinal strain rate (/sec)	− 1.12 ± 0.25	− 1.03 ± 0.25	0.011
Left ventricular diastolic longitudinal strain rate (/sec)	1.15 ± 0.26	0.95 ± 0.24	< 0.001

## Discussion

Cardiac events are a common finding in IIM patients and a major cause of mortality [3, 4]. Early diagnosis of cardiac involvement in inflammatory muscle diseases is therefore necessary to allow for early detection of patients at risk [5, 6]. Significantly lower myocardial deformation parameters in IIM patients in comparison to a healthy control cohort were observed in our study. Moreover, LVEF was not significantly reduced, suggesting that a change in myocardial deformation can be observed before decrease of ejection fraction. Therefore, myocardial deformation parameters may serve to detect high risk IIM patients in good time before cardiac events occur.

We used CMR feature tracking and not echo speckle tracking for the strain analysis in our trial, since CMR is the current gold standard for the evaluation of ventricular volumes, the global and regional function. This is due to its ability to image 3D volumes unaffected by the patients habitus. For this reason, CMR has a superior inter- and intraobserver variability compared to echo. It is possible but yet not proven that CMR is thereby better than echo in the question of deformation parameters. In a current study by Pryds et al. speckle tracking echocardiography and feature tracking CMR have shown a good correlation. Both methods are thereby not interchangeable at custom, since the global longitudinal strain was measured higher and the global radial and circumferential strain lower with speckle tracking echocardiography [15]. Otherwise, CMR has the advantage of the possibility of a non-invasive tissue characterization with LGE and increasingly with parametric mapping. Deformation parameters seem to correlate with LGE in some entities but cannot replace it according to current knowledge [16]. At least, speckle tracking echocardiography can fail due to low image quality and requires an experienced examiner, while—of course—CMR is more expensive and not as widely available.

In other disease entities, deformation parameters have been shown to have a high diagnostic and predictive value. Doerner and colleagues found that especially in patients with preserved left ventricular function and suspected myocarditis, left ventricular strain parameters in combination with the Lake-Louise-Criteria improve diagnostic performance. Patients with myocarditis had a significantly reduced LVGLS in comparison to healthy volunteers (− 17.2 ± 4.9% vs. − 13.3 ± 6.2%, p < 0.001) [17]. We showed a quite similar difference in longitudinal strain between IIM patients and healthy counterparts.

Another recent trial showed that LVGLS was the only independent predictor of functional recovery in patients with myocarditis [18]. A high predictive value of LVGLS was also shown for other myocardial diseases like Takotsubo cardiomyopathy, ST-segment elevation myocardial infarction and chemotherapy induced cardiomyopathy [12, 13, 19]. Future investigations are warranted to evaluate the relationship between early changes in deformation parameters and clinical outcome in IIM patients.

For no obvious reason we had a difference in RVEF favoring the IIM-study group. We cannot explain whether this is due to an unknown confounder or small sample size. In fact, the values were not pathological [20] and there were no clinical signs of right ventricular insufficiency neither in the IIM nor in the control group.

Another potentially important diagnostic parameter for different cardiomyopathies is presence of LGE, which is a marker of fibrosis and consequently of irreversible myocardial remodeling. It is a common finding, *inter alia*, in infectious myocarditis [8]. As previously shown, a pathological LGE pattern is also often present in IIM patients [11]. In our cohort, LGE presence was associated with reduction in LVEF, LVGLS and RVGLS. Lee et al. have shown a correlation between LGE and occurrence of cardiac events in patients with acute myocarditis [21]. Such relationships are also conceivable for IIM patients and should be subject of further investigation.

In our study cohort, a significant decrease of LVEF and all strain parameters were observed between baseline and follow-up. A recent meta-analysis could exclude a potential correlation between age and deformation parameters [22]. Hence, a subclinical deterioration of myocardial function over time can be excluded. Observed worsening of functional parameters in our study Is consequently due to the underlying disease. Thus, a single CMR examination may not provide enough information to detect patients at risk. A follow-up CMR exam can provide information about functional worsening and thereby contribute to therapeutic decision as therapy escalation.

### Study limitations

The main limitation of this study is the small sample size. This is essentially due to the fact that IIM are rare diseases according to the Rare Diseases Act of 2002 of the United States. In fact, our study population is large compared to others found in literature. Another limitation is the unicentric study design. Our data should be validated in larger cohorts of multicentric studies. Furthermore, our study was not designed to examine the impact of imaging findings on clinical decision-making and patient outcome. Whether a change in immunosuppressive therapy because of MR-findings has an influence on clinical outcome should be investigated in a prospective randomized trial.

## Conclusions

Cardiac involvement in IIM was frequently identified. We have shown that CMR including LGE and feature tracking provides additional information to classical ejection fraction measurements and may improve diagnostic work-up of IIM patients in future.

## Data Availability

The datasets used and/or analyzed during the current study are available from the corresponding author on reasonable request.

## Abbreviations

CMR: Cardiovascular magnetic resonance
IIM: Idiopathic inflammatory myopathy
LGE: Late gadolinium enhancement
LV: Left ventricular
LVEDVi: Left ventricular end-diastolic volume index
LVEF: Left ventricular ejection fraction
LVGCS: Left ventricular global circumferential strain
LVGLS: Left ventricular global longitudinal strain
LVGRS: Left ventricular global radial strain
RVEDVi: Right ventricular end-diastolic volume index
RVEF: Right ventricular ejection fraction
RVGLS: Right ventricular global longitudinal strain
